# Positive regulation of TAZ expression by EBV-LMP1 contributes to cell proliferation and epithelial-mesenchymal transition in nasopharyngeal carcinoma

**DOI:** 10.18632/oncotarget.13775

**Published:** 2016-12-02

**Authors:** Jiang He, Feiyu Tang, Liyu Liu, Lin Chen, Jiang Li, Danming Ou, Lu Zhang, Zhi Li, Deyun Feng, Wenzheng Li, Lun-Quan Sun

**Affiliations:** ^1^ Center for Molecular Medicine, Xiangya Hospital, Collaborative Innovation Center for Cancer Medicine, Central South University, Changsha, 410008, China; ^2^ Key Laboratory of Molecular Radiation Oncology Hunan Province, Changsha, 410008, China; ^3^ Department of Pathology, Xiangya Hospital, Central South University, Changsha, 410008, China; ^4^ Department of Radiology, Xiangya Hospital, Central South University, Changsha, 410008, China

**Keywords:** EBV, LMP1, TAZ, nasopharyngeal carcinoma

## Abstract

The Epstein-Barr virus latent membrane protein 1 (LMP1) is an integral membrane protein. LMP1 has been reported to activate the NF-κB and mitogen-activated protein kinase pathways. However, these effects alone are unable to account for the profound oncogenic properties of LMP1. TAZ is one of the nuclear effectors of Hippo-related pathways and highly expressed in many human tumors. Here, we reported that TAZ was frequently expressed in LMP1-positive nasopharyngeal carcinoma. In NPC cell lines, we showed that LMP1 promoted TAZ expression. Gelsolin is an important inhibitor of TAZ activity. Our studies showed that LMP1 interacted with gelsolin, resulting in inhibition of Lats1/2 phosphorylation and improvement of TAZ stability. Furthermore, we revealed that TAZ is important for LMP1-mediated cell proliferation, cancer stem cell-like properties and epithelial-mesenchymal transition. These findings provide new insights into the carcinogenic roles of LMP1 and contribute to further understanding of its oncogenic mechanism.

## INTRODUCTION

Epstein-Barr virus (EBV) is a ubiquitous human herpesvirus which is involved in many human malignancies, such as nasopharyngeal carcinoma (NPC), Burkitt's lymphoma, T-cell lymphoma, gastric carcinoma, and invasive breast cancer [1, 2]. Constant EBV infection can efficiently transform resting B cells into permanently growing lymphoblastoid cell lines *in vitro*. The latent membrane protein 1 (LMP1), a 62-kDa integral membrane protein, is one of the most important oncogenic proteins of human DNA tumor virus EBV. Driven by an Ig heavy chain promoter/enhancer, the LMP1 expression resulted in lymphomas with high incidence in transgenic mice, indicating that LMP1 alone has a transforming potential [3, 4].

LMP1 protein has three domains, including a short NH2-terminal sequence (amino acids 1–23), six hydrophobic membrane-spanning domains that mediate self-aggregation and oligomerization (amino acids 24–186), and C-terminal activation regions 1 and 2 (CTAR1 and 2, amino acids 187–386) located in the cytoplasm that possesses most of the signaling activity of the molecule [5]. CTAR1 and CTAR2 have been reported to be involved in the induction of NF-κB transcription factor pathway [5]. The ability that LMP1 immortalizes and transforms cells is most likely associated with simultaneously controlling cellular signaling pathways that block apoptosis or mediate proliferative, growth factor-like effects. In addition, LMP1 could induce a cancer progenitor cells (CPC)-like phenotype in epithelial cells, suggesting that LMP1-induced phenotypic changes may contribute to the development of NPC [6]. Thus, the mechanism by which LMP1 immortalizes and transforms cells is complexed and some of the key questions remain to be elucidated.

TAZ is an important member of Hippo pathway. The Hippo pathway composed of Hpo, Sav, Wts, Mats, and Yki, is highly conserved throughout evolution. Their mammalian orthologs are mammalian sterile 20-like 1/2 (MST1/2, also called STK4/3), Salvador (SAV1), large tumor suppressor homolog 1/2 (LATS1/2), MOB kinase activator 1A/B (MOB1a/b), and Yes-associated protein (YAP)/transcriptional co-activator with PDZ binding motif (TAZ, also called WWTR1), respectively [7–11]. TAZ overexpression is found in many primary tumors and could stimulate many biological processes, including cell proliferation and organ size [12, 13]. Activated MST1/2 can then directly phosphorylate LATS1 and LATS2, negatively regulating the oncoprotein and transcriptional coactivator Yorkie (Yki) or its mammalian orthologs Yap and TAZ [14, 15]. The inhibition of Hippo pathway increases TAZ expression. Many studies have shown that the loss of Hippo signaling or overexpression of YAP/TAZ is sufficient to causes overgrowth of various organs and tumor formation in the liver, skin and colon of mice [16–22].

The actin cytoskeleton is not only important in maintaining cell morphology, but also plays important roles in regulating cell proliferation and differentiation. Many studies have shown that the actin cytoskeleton is involved in regulation of cell proliferation through the Hippo pathway in both flies and mammals. In mammalian tissue culture, Yap and TAZ activity and subcellular localization was regulated by changes in cell morphology and the actin cytoskeleton [23–25]. Knockdown of different regulators of the actin cytoskeleton, including cofilin, CapZ and gelsolin, increased TAZ activity. For instance, loss of actin-capping proteins in Drosophila resulted in Yki activation and tissue overgrowth [26, 27]. In addition, pharmacological inhibition of microtubules (MTs) and F-actin increased Lats kinase activity and its ability to phosphorylate TAZ *in vitro* [27].

While the signaling balance between the tumor suppression of Hippo pathway and the oncogenic drivers or promoters of oncogenic viruses remains to be fully determined, understanding of these processes may help to explain mechanisms of oncogenic viral signaling events and DNA virus associated diseases. Here, we demonstrated that EBV-LMP1 inhibited LATS1/2 phosphorylation and increased TAZ stability. The inhibition of Hippo pathway was mediated by cytoskeletal remodeling by LMP1 interaction with gelsolin and led to the increased cell proliferation, EMT and cancer stem cell (CSC)-like properties. Thus, the present study suggests an important mechanism of EBV-induced oncogenesis.

## RESULTS

### LMP1 increased TAZ expression

**Figure 1:**
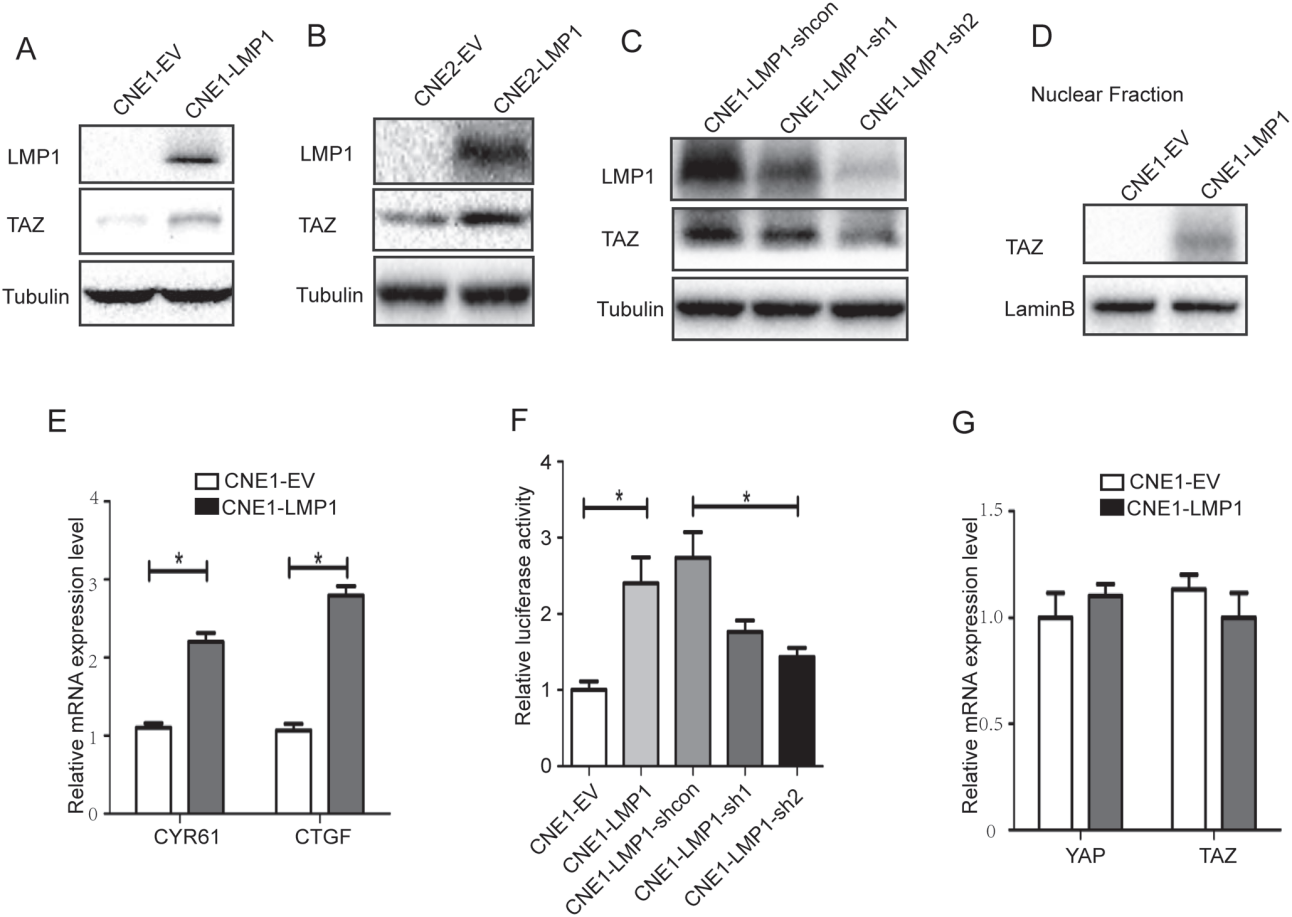
Effect of LMP1 on TAZ expression **A** and **B**. The protein from CNE1-EV/CNE1-LMP1 and CNE2-EV/CNE2-LMP1 cells was analyzed by western blot using antibodies against TAZ, LMP1, and Tubulin. **C**. The protein from CNE1-LMP1 cells with LMP1 knocking down was analyzed by western blot using antibodies against TAZ, LMP1 and Tubulin. **D**. CNE1-EV/CNE1-LMP1 cells were fractionated for nuclear extracts. Protein was separated by SDS-PAGE and blotted using antibodies against TAZ and Lamin B. **E**. mRNA levels of Cyr61 and CTGF (TAZ regulated targeted genes) were analyzed by real-time PCR in CNE1-EV, CNE1-LMP1. **F**. Luciferase activity of the YAP/TAZ reporter in CNE1-EV, and CNE1-LMP1, CNE1-LMP1-shcon, CNE1-LMP1-sh1 and CNE1-LMP1-sh2 cells. **G**. mRNA levels of YAP and TAZ were analyzed by real-time PCR in CNE1-EV, CNE1-LMP1 cells. All experiments were performed at least 3 times and data were expressed as mean ± SD. *P < 0.05 between assigned groups. EV, empty vector; sh, short hairpin RNA; shcon, short hairpin RNA control.

The previous reports demonstrated that LMP1 induced actin filament remodeling [28]. The actin cytoskeleton is important for regulation of Hippo pathway. These evidences prompted us to investigate whether LMP1 could elevate TAZ expression by suppression of Hippo pathway. Western blot were performed to determine whether TAZ was upregulated by LMP1. Cells included LMP1-positive cells, CNE1-LMP1 and CNE2-LMP1, and their control cells stably transfected with empty vector, CNE1-EV and CNE2-EV. The results showed that the level of TAZ protein increased in CNE1-LMP1 and CNE2-LMP1 cells compared with their control cells (Figure 1A and 1B). To further confirm LMP1 promoted TAZ expression, we used a lenti-shRNA approach to target LMP1. Then western blot was performed to determine whether TAZ expression was elevated. We found the LMP1-targeted CNE1-LMP1 cells reduced TAZ expression (Figure 1C). CTGF and Cyr61 are the target genes of TAZ [29]. To further study the effect of the LMP1-induced TAZ expression on the down-stream targets of TAZ, the CTGF and Cyr61 mRNAs level were determined by real-time PCR. As shown in Figure 1E, LMP1 could stimulate the mRNA expression of CTGF and Cyr61.

Next, we tested the effect of LMP1 on nuclear localization of TAZ. Western blot analysis of the nuclear fraction with TAZ antibody indicated that LMP1 increased the nuclear localization of TAZ (Figure 1D). Since LMP1 promoted the TAZ nuclear localization, LMP1 might enhance the TAZ activity. Transcriptional activity of the TAZ was determined by a luciferase reporter assay in CNE1-EV cells and CNE1-LMP1 cells. We found LMP1 elevated TAZ transcriptional activity while knockdown of LMP1 diminished TAZ transcriptional activity (Figure 1F). To test whether LMP1 increased TAZ expression at the transcriptional level, the level of mRNA of in CNE1-LMP1 and CNE1-EV cells was extracted. The level of YAP/TAZ mRNA was detected by real-time PCR. As shown in Figure 1G, LMP1 did not significantly impact on the mRNA level of TAZ. Thus, these results suggested that the elevation of TAZ protein level by LMP1 was not via induction of its transcription, but by an increased synthesis of the protein or its stabilization.

### LMP1 enhanced TAZ stability

**Figure 2:**
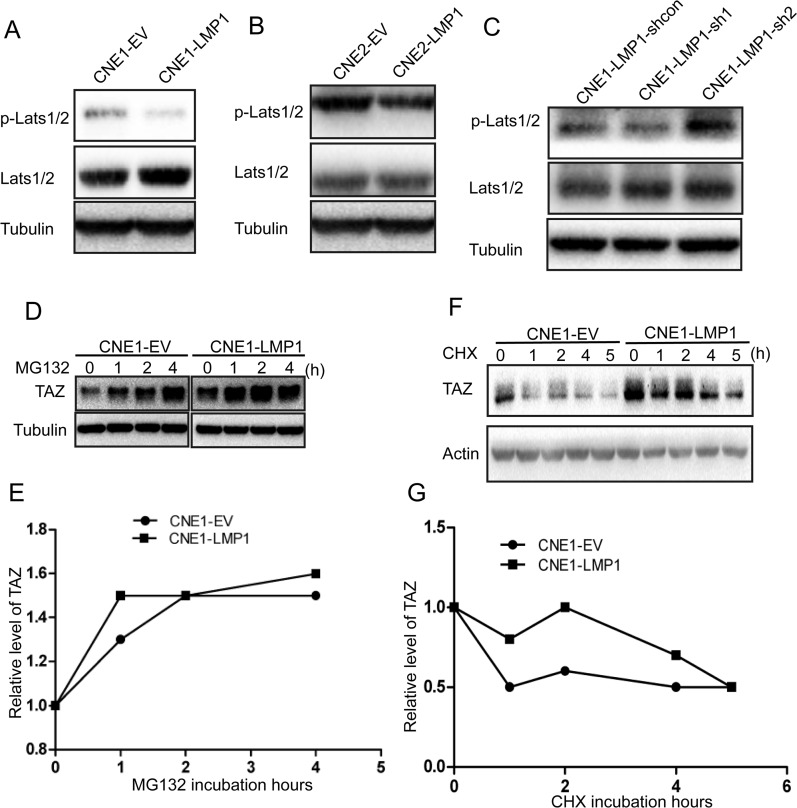
Effect of LMP1 on TAZ stability **A** and **B**. The protein from CNE1-EV/CNE1-LMP1 and CNE2-EV/CNE2-LMP1 cells was analyzed by western blot using antibodies against p-Lats1/2, Lats1/2 and Tubulin. **C**. The protein from CNE1-LMP1 cells with LMP1 knocking down was analyzed by western blot using antibodies against p-Lats1/2, Lats1/2 and Tubulin. **D**. CNE1-EV/CNE1-LMP1 cells that were treated with MG132 for different time intervals were analyzed by western blotting. Equal amounts of protein were subjected to western blot analysis, as determined by comparing amounts of tubulin. **E**. Densitometry of TAZ levels after MG132 treatment was plotted. **F**. TAZ proteins from CNE1-EV and CNE1-LMP1 cells treated with 20 μM cycloheximide (CHX) at different time intervals were analyzed by western blotting. **G**. Densitometry results for TAZ from CNE1-EV and CNE1-LMP1 cells after cycloheximide treatment were plotted.

Activated LATS1/2 phosphorylates the WW-domain of the transcriptional coactivator TAZ, resulting in TAZ degradation [10, 16, 30]. We showed that LMP1 elevated the level of TAZ and promoted TAZ nucleus translocation (Figure 1E) Therefore, we hypothesized that LMP1 might inhibit LATS1/2 activation, leading to the enhancement of TAZ stability. To prove this hypothesis, protein was extracted from CNE1-LMP1 and CNE-EV cells. Western blot was performed to determine LATS1/2 phosphorylation level using a phosphor-LATS1/2 antibody. The result indicated that LATS1/2 phosphorylation level was significantly downregulated in CNE1-LMP1 and CNE2-LMP1 cells compared with CNE1-EV and CNE2-EV cells, respectively (Figure 2A and 2B). Knockdown of LMP1 resulted in an increase of LATS1/2 phosphorylation level (Figure 2C). These data suggested that LMP1 can suppress the activation of LATS1/2.

To further investigate whether the inhibition of Lats1/2 phosphorylation by LMP1 could promote the stability of TAZ, we examined the stability of TAZ in the presence or absence of LMP1. Firstly, we treated cells with MG132, a proteasome inhibitor, and then performed western blot analysis at different time points. The result showed that the TAZ protein level gradually increased and reached a maximum level at 4 h after MG132 treatment in LMP1-negative cells. However, TAZ was much more stable in LMP1-positive cells than that in LMP1-negative cells, as their protein levels increased and reached a maximum 1h after MG132 treatment (Figure 2D and 2E). Next, we incubated cells with cycloheximide (CHX), a potent translation inhibitor, and then performed Western blot analysis at different time points. The half-life of TAZ was shown to be in CNE1-EV cells is 1 h and 2.5h in CNE1-LMP1 cells. At all of the time points, levels of the TAZ protein were significantly higher in LMP1-positive cells than that in LMP1-negative cells (Figure 2F and 2G). These results suggested that LMP1 could increase TAZ stability.

### LMP1 inhibited Lats1/2 phosphorylation to improve TAZ stability through gelsolin

**Figure 3:**
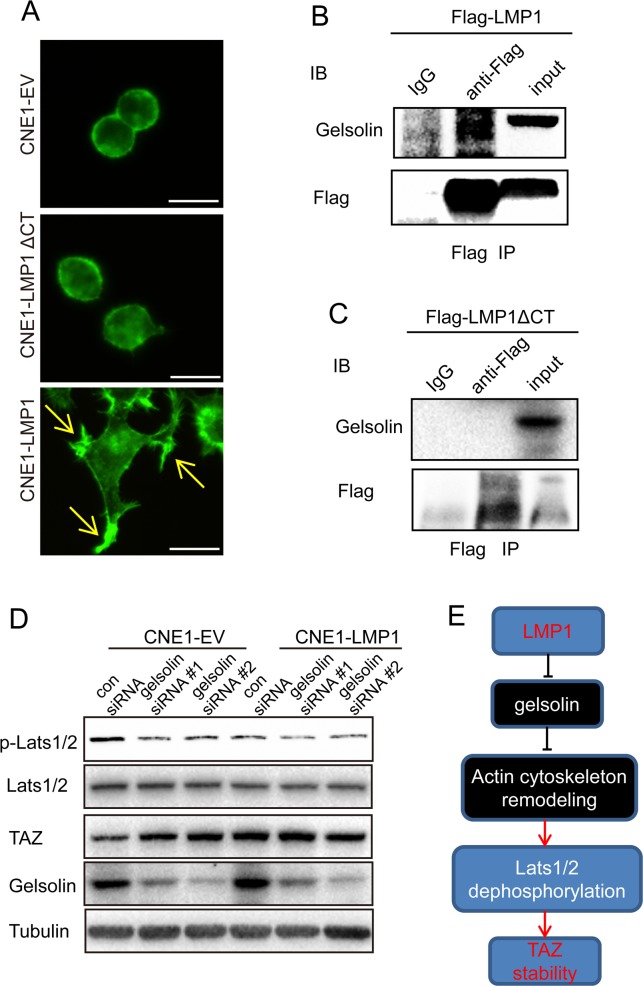
LMP1 increases TAZ stability through interaction with gelsolin **A**. Induction of F-actin rearrangement by LMP1. CNE1-EV, CNE1-LMP1 and CNE1-LMP1-ΔCT (deletion of the C-terminus) cells were grown on poly-L-lysine-coated coverslips overnight. Cells were fixed, and stained with a FITC-conjugated phalloidin (Green). The expression of LMP1 led to formation of the microspike-like actin structures (filopodia) at the plasma membrane (arrows). Images were acquired using a Leica microscope as detailed in Materials and Methods. Scale bars, 50 mm. Transfection of an empty vector had no effect on actin organization. **B** and **C**. LMP1 or LMP1-ΔCT interacts with gelsolin. Lysates from HEK 293T cells transfected with the indicated combinations of WT or truncation mutants of Flag-tagged LMP1 were subjected to anti-Flag IP followed by anti-Flag and anti-gelsolin IB. **D**. Effect of gelsolin on LMP1-induced TAZ signaling. CNE1-EV/CNE1-LMP1 cells were analyzed by western blot after transfection with gelsolin siRNA using antibodies to p-Lats1/2, Lats1/2, TAZ and tubulin. **E**. Proposed mechanism for LMP1 promotion of TAZ stability.

As mentioned above, LMP1 inhibited Lats1/2 phosphorylation. In addition, it has been reported that the actin cytoskeleton could modulate the phosphorylation of LATS1/2 [30]. These evidences suggest that LMP1 might remodel actin cytoskeleton and thus inhibit Lats1/2 phosphorylation, resulting in TAZ stability. To verify the hypothesis, we firstly determined whether LMP1 induced actin filament remodeling. CNE1-LMP1 and CNE1-EV cells were fixed and stained with a FITC-conjugated phalloidin. Cellular images were acquired using confocal microscopy. As shown in Figure 3A, the expression of LMP1 led to morphologic changes in CNE1-LMP1 cells, which formed the microspike-like actin structures (filopodia) at the plasma membrane and the actin bundles at the perinuclear regions compared with CNE1-EV cells. However, a mutant form of LMP1 (C-terminus-deleted, LMP1ΔCT) failed to induce any features of the actin remodeling in CNE1 cells. These results showed that LMP1 contribute to actin cytoskeleton remodeling. In addition, knockdown of gelsolin also resulted in formation of microspike-like actin structures in CNE1-EV cells. In contrast, knockdown of gelsolin did not lead to obvious change of actin substructures (microspike-like actin structures) in CNE1-LMP1 cells (Supplementary Figure S1). These results showed that gelsolin is involved in LMP1-induced actin cytoskeleton remodeling.

It is reported that gelsolin is an important inhibitor for actin cytoskeleton remodeling [31, 32]. Previous studies also showed that gelsolin was essential for limit of TAZ activity in the cells experiencing low mechanical stresses, such as contact inhibition [31]. These evidences, together with our findings that LMP1 induced actin cytoskeleton remodeling through gelsolin and enhanced TAZ stability, suggest that the expression of LMP1 might result in loss of gelsolin function and actin cytoskeleton remodeling, and thus increasing TAZ stability. To confirm the hypothesis, we examined whether LMP1 could interact with gelsolin. HEK293T cells were transfected with plasmid encoding flag-tagged full-length LMP1 and LMP1ΔCT. Flag-tagged LMP1 was immno-precipitated with flag antibody. Immuno-blotting results showed that LMP1 physically interacted with gelsolin through C-terminus (Figure 3B and 3C). Next, we investigated whether LMP1 could increase Lats1/2 phosphorylation and TAZ expression after gelsolin knockdown in CNE1-EV and CNE1-LMP1 cells. In CNE1-EV cells, the TAZ expression was markedly up-regulated, whereas Lats1/2 phosphorylation was downregulated after knocking down gelsolin. In contrast, in the presence of LMP1, gelsolin knockdown only had marginal effect on the LATS1/2 phosphorylation and TAZ expression (Figure 3D). These results thus suggested a model in which LMP1 remodeled actin cytoskeleton through gelsolin, thus inhibiting Lats1/2 phosphorylation and increasing TAZ stability (Figure 3E).

### LMP1 enhanced cell proliferation and CSC-like properties by TAZ

**Figure 4:**
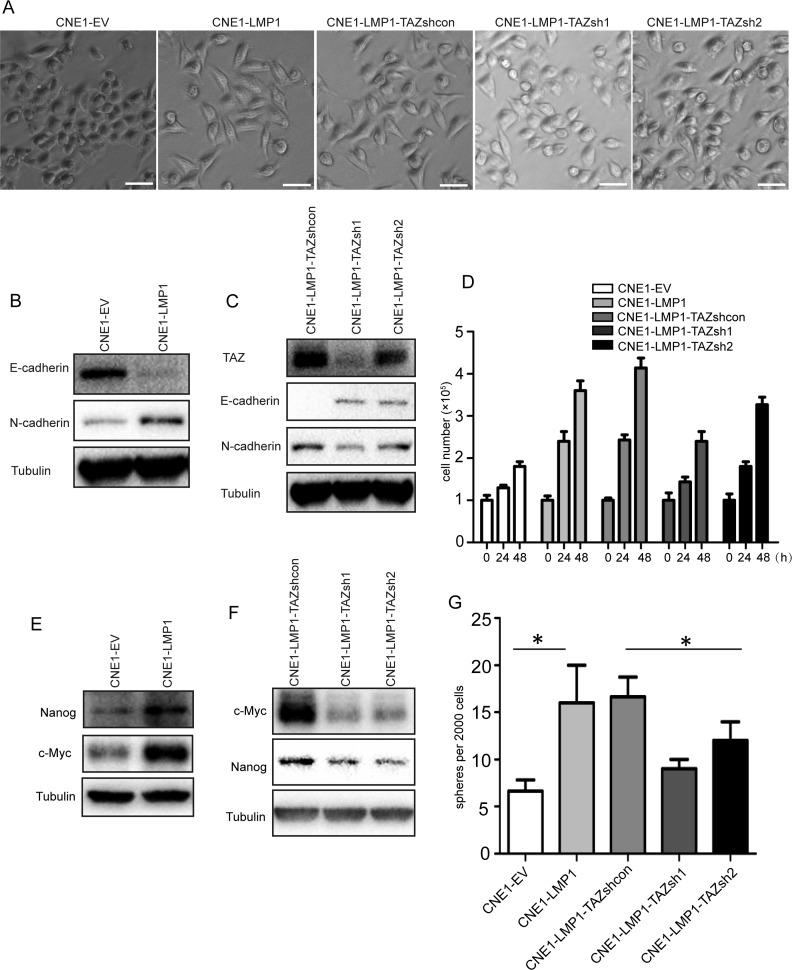
LMP1 enhances cell proliferation and cancer stem cell-like properties by TAZ **A**. LMP1 regulation of cell phenotype by TAZ. Representative phase-contrast images are presented for the CNE1-EV, CNE1-LMP1, TAZ-depleted CNE1-LMP1 and CNE1-LMP1-TAZ shcon cells. TAZ-depleted CNE1-LMP1 cells are LMP1-overexpressed cells with TAZ knocking-down by shRNA (CNE1-LMP1-TAZ sh1 and CNE1-LMP1-TAZ sh2) and CNE1-LMP1-TAZ shcon cells are the corresponding control cells. Scale bars, 50 μm. **B** and **C**. LMP1 regulation of EMT genes expression via TAZ. Lysates from the control CNE1, CNE1-LMP1 and TAZ-depleted CNE1-LMP1 cells were analyzed by western blot using antibodies to E-cadherin, N-cadherin and tubulin. **D**. LMP1 promotion of cell proliferation via TAZ. Cell proliferation was determined by cell counting in the CNE1-EV, CNE1-LMP1 and TAZ-depleted CNE1-LMP1 cells. **E** and **F**. LMP1 regulates stemness gene expression via TAZ. Lysates from the CNE1-EV, CNE1-LMP1 and TAZ-depleted CNE1-LMP1 cells were analyzed by western blot using anti-Myc, anti-nanog and tubulin antibodies. **G**. Quantification of the spheres formed in shcon or shTAZ CNE1-LMP1 cell cultures. All experiments were performed at least 3 times and data were expressed as mean ± SD. *P < 0.05 between assigned groups. sh, short hairpin RNA; shcon, short hairpin RNA control.

TAZ has been suggested to be involved in positive regulation of cell proliferation and survival coupled with the acquisition of CSC characteristics and epithelial to mesenchymal transition (EMT) [33, 34]. To test whether LMP1 could promote EMT through TAZ, we used shRNA to deplete TAZ in CNE1-LMP1 cells and determined whether the morphology changed. The result showed that knockdown of TAZ in CNE1-LMP1 cells caused the morphological changes from epithelial to mesenchymal phenotypes (Figure 4A). To test the effect of LMP1 on the expression of the EMT genes, western blot was performed to determine the levels of E-cadherin and N-cadherin. The results indicated that LMP1 increased N-cadherin expression and decreased E-cadherin expression, and knockdown of TAZ resulted in the downregulation of E-cadherin and N-cadherin (Figure 4B and 4C). These results indicated that LMP1 could promote EMT of NPC cells through TAZ. Next, we further investigated whether LMP1 could drive cell proliferation through TAZ. Firstly, we found that LMP1 expression resulted in increased cell number, whereas stable knockdown of LMP1 resulted in decreased cell number in CNE1-LMP1 cells (Supplementary Figure S2). Then, we generated the CNE1-LMP1 cell lines with stable knockdown of TAZ. As expected, stable knockdown of TAZ resulted in decreased cell numbers (Figure 4D).

In consideration of previous reports that TAZ overexpression or Hippo pathway inhibition promoted cancer cell stemness [29, 33], we further examined whether LMP1 enhanced CSC stemness via TAZ. To test the effect of LMP1 on stemness genes expression, western blot was performed to determine the levels of c-Myc and Nanog in CNE1-EV and CNE1-LMP1 cells. The result showed that LMP1 increased the expression of c-Myc and Nanog and knockdown of TAZ caused significant down-regulation of c-Myc and Nanog (Figure 4E and 4F). In addition, CNE1-LMP1 cells formed more spheres than CNE1-EV cells, while knockdown of TAZ in CNE1-LMP1 cells led to significantly less spheres than that in the control cells (Figure 4G). Collectively, these results indicated that TAZ was important for LMP1 to promote cancer cell proliferation and stemness.

### LMP1 promoted TAZ nuclear localization in NPC and gastric cancer patient samples

**Figure 5:**
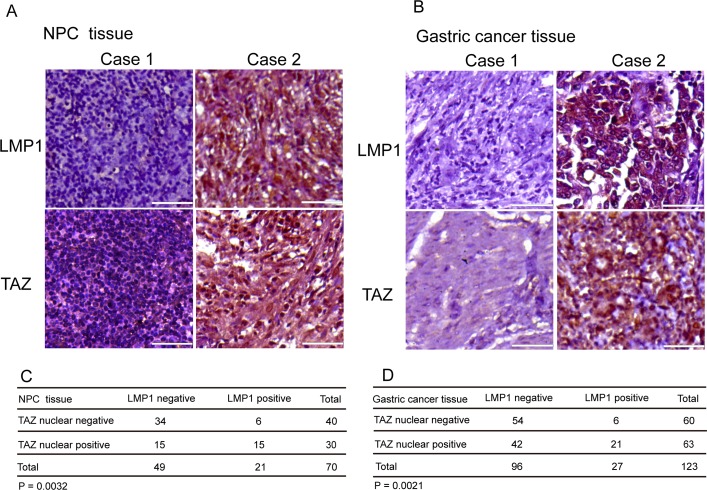
Association of TAZ upregulation with LMP1 in human NPC and gastric cancer samples **A** and **B**. Immunohistochemical staining of LMP1 and TAZ in the representative slides of LMP1-negative and LMP1-positive NPC and gastric cancer specimens. Brown staining indicates positive immunoreactivity. Scale bars, 50 μm. **C** and **D**. Correlation between LMP1 and TAZ protein levels in human NPC (C) and gastric cancer (D) tissues. Statistical significance in C and D was determined by χ^2^ test.

Previous reports have shown that the Hippo pathway was deregulated and TAZ was activated in a broad range of human cancers, which was often correlated with poor patient prognosis, including lung, colorectal, ovarian, liver and prostate cancers [12, 16, 22, 30, 35–40]. The TAZ nuclear localization has been frequently observed in malignant cells, such as 15% of ovarian cancers [41], 65% of non-small-cell lung cancers [42] and approximately 60% in hepatocellular carcinomas [40]. LMP1 is associated with NPC and gastric cancer [43]. To determine the clinical relevance of regulation of TAZ by LMP1 in patients, we performed immunohistochemical staining of TAZ and LMP1 on the NPC and gastric cancer tissues. Notably, nuclear localization of TAZ was observed in 71.4% (15 of 21) and 77.8% (21 of 27) of LMP1-positive NPC and gastric cancer tissues separately, whereas only 30.6% (15 of 49) and 43.8% (42 of 96) of LMP1-negative NPC and gastric cancer tissues exhibited positive nuclear staining of TAZ respectively (Figure 5C and 5D). It suggested that nuclear localization of TAZ was strongly associated with LMP1 in human NPC and gastric cancer tissues. A high correlation between the LMP1 and TAZ levels observed in NPC (n=70; *P* < 0.05; Figure 5C) and gastric cancer (n=123; *P* < 0.05; Figure 5D). The representative images of LMP1+/− and TAZ+/− NPC and gastric cancer tissues were shown respectively in Figure 5A and 5C. Together, these data suggested that expression of LMP1 may contribute to the stability and nuclear localization of TAZ in tumor tissues, which promoted cancer progression.

## DISCUSSION

LMP1 is a constitutively active pseudo-receptor that acts as a key player during B cell immortalization [44]. As LMP1 is the only EBV-encoded protein with the characteristics of a classical oncogene, full understanding of the signaling capacity of LMP1 is crucial to defining its role in EBV-induced oncogenesis. However, previous work has focused on the contribution of the NF-kB and mitogen-activated protein kinase pathways to LMP1-induced effects [44]. LMP1 is reported to activate the NF-κB and mitogen-activated protein kinase (MAPK) pathways [45]. A recent study in fly suggested that the MAPK pathway was a new upstream branch of the Hippo pathway, which presented a possible link between MAPK and Hippo pathways [46]. However, whether or not this link is present in human cancer cells remains to be verified. Considering the facts that LMP1 can both activate the MAPK pathway and increase TAZ activity as shown in our present study, it is possible that LMP1 synergistically promotes cell transformation through MAPK and TAZ.

The Hippo signaling has been emerging as an efficient pathway for growth control and tumor suppression. The loss of Hippo activity that hyper-activates YAP and TAZ in mouse models causes overgrowth of various organs such as the liver and heart [16, 20, 22]. Indeed, there is considerable evidence that abnormal Hippo signaling is associated with several human cancers, including the development of cancer in the liver, skin and intestine [16–22]. Elevated levels and nuclear localization of YAP and TAZ have been reported in the majority of solid cancers, which suggests that there is a widespread deregulation of Hippo signaling in human neoplasia. It has been shown that the attenuation of Hippo signaling or overexpression of YAP/TAZ is sufficient to promote tumor formation in mice. The exact mechanisms involved in the transformation of normal cells to malignant cells by deregulation of YAP and TAZ are yet to be known, but are likely to involve the enhanced cell proliferative and survival capacity. Particularly, YAP and TAZ have been shown to participate in promotion of some of the cancer hallmarks, such as EMT, CSC stemness, drug resistance and inhibition of senescence [33]. In agreement with these reports, we showed that LMP1 can promote cell proliferation, EMT and CSC-like properties through TAZ and provided some explanations for the LMP1-mediated resistance to radiotherapy [47].

Many studies have shown that the actin cytoskeleton is required for the morphological and cell-cycle modulations that accompany cell-cell adhesion and cellular binding with extracellular matrix (ECM) [48]. Studies embracing the actin cytoskeleton and cell growth have shown that the Rho family of small GTPases is involved in regulation of cell growth through the actin cytoskeleton remodeling. More recently, the studies have shown that the actin cytoskeleton is involved in regulating cell proliferation through the Hippo pathway in both flies and mammals. In mammalian tissue cultures, Yap activity and subcellular localization is regulated by the changes in cell morphology and the actin cytoskeleton, although there are currently some debates about whether the core Hippo signaling components are involved [12–14]. For example, Wada et al. and Zhao et al. showed that disrupting the actin cytoskeleton inhibited Yap activity in a Lats-dependent fashion [13, 49]. Here, we showed that LMP1 can stimulate actin cytoskeleton via an interaction with gelsolin, which is necessary for the cellular events that require cytoskeletal remodeling [50]. Mechanistically, LMP1 interacts with gelsolin through the CTAR domain, possibly leading to disrupting the actin-gelsolin complex, which results in the actin cytoskeleton remodeling and Hippo pathway inhibition.

**Figure 6:**
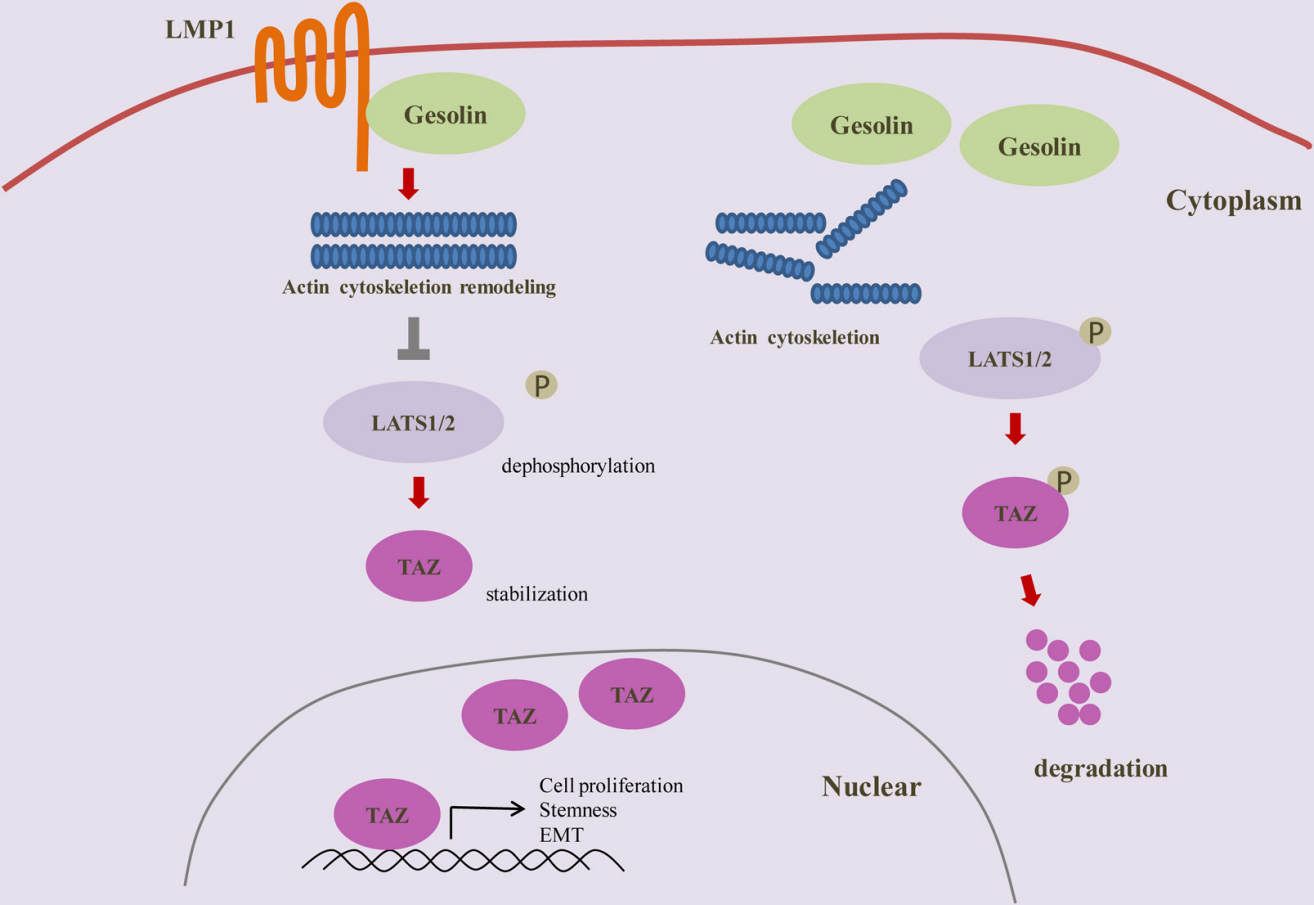
Schematic representation of positive regulation of TAZ by EBV-LMP1 in nasopharyngeal carcinoma LMP1 interaction with gelsolin contributes to actin cytoskeleton remodeling, and thus inhibits Lats1/2 phosphorylation and enhances TAZ stability, causing cell proliferation, EMT and stemness.

In conclusion, the present study showed that EBV-LMP1 promoted actin cytoskeleton remodeling through interaction with gelsolin, and thus inhibited LATS1/2 phosphorylation and enhanced TAZ stability. TAZ is an important transcriptional factor that controls cell proliferation and organ size in response to changing cell density. Here, we found TAZ is important for LMP1-mediated cell proliferation, cancer stem cell-like properties and EMT phenotypes. These findings provided further insights into the oncogenic roles of EBV-LMP1 and revealed a potential new signaling pathway for the EBV-induced oncogenesis (a model is presented in Figure 6).

## MATERIALS AND METHODS

### Cell lines and culture

CNE1-LMP1 and CNE2-LMP1 were constructed from LMP1-negative NPC cell lines, which were stably transfected with a pLV-LMP1 plasmid and treated with 2 μg/ml blasticidin for 7days. LMP1 knocked-down cell lines (CNE1-LMP1-sh1 and CNE1-LMP1-sh2) were constructed with pLVX-LMP1shRNA lentivirus transducing CNE1-LMP1 cells and treated with 1 μg/ml puromycin for 3 days. CNE1-LMP1-ΔCT cells were constructed from CNE1 cells, which were stably transfected with a pLV-LMP1-ΔCT (deletion of the C-terminus) plasmid and treated with 2 μg/ml blasticidin for 7 days. These cell lines were maintained in RPMI1640 (Gibco, New York, USA). The human embryonic kidney (HEK) cell line 293T was maintained in DMEM (Invitrogen, Carlsbad, CA, USA). Both media were supplemented with 10% fetal bovine serum (FBS, Gibco, New York, USA), 100 U/ml penicillin, and 100 mg/ml streptomycin (Invitrogen, Carlsbad, CA, USA), and all of the cells were incubated in a humidified atmosphere of 5% CO2 at 37°C.

### Plasmids and shRNA

The pSG5- LMP1 expression vector was a gift from Professor Ya Cao of Cancer Research Institute, Central South University. LMP1 and LMP1-ΔCT (deletion of the C-terminus) cDNA from pSG5- LMP1 plasmid was further cloned into CMV2B Flag vector and pLV-cDNA vector. The targeting sequences of siRNA/shRNA for LMP1 and TAZ were listed in Supplementary Table S2. The shRNAs were cloned into pLVX-shRNA vector and subjected to further lentiviral production.

### RNA extraction and quantitative RT-PCR

Total RNA was extracted from the control CNE1 and CNE1-LMP1 cells using TRIzol reagent (Invitrogen, Carlsbad, CA, USA). One microgram of RNA was treated with 1 unit of DNase for 1 h at 37°C, and cDNAs were synthesized using Reveraid First Strand cDNA synthesis kit (Thermo Scientific, USA). qRT-PCR was performed using the SYBR Green PCR Master Mix (Bio-Rad, Hercules, CA, USA) and the CFX-96 Real-time PCR System (BioRad, Hercules, CA, USA). The primer sequences were listed in Supplementary Table S1.

### Protein preparation and western blotting

Total protein was extracted using RIPA Lysis Buffer (Pierce Biotechnologies Inc., Rockford, USA) containing complete protease inhibitor cocktail (Roche, Basel, Switzerland) and phosphatase inhibitor (Roche, Basel, Switzerland), following a wash with cold PBS for twice. The concentration of the protein in the supernatant was measured using the BCA Protein Assay Kit (Pierce Biotechnologies Inc., Rockford, USA). Equal amounts of protein were boiled for 5 min in 6× sodium dodecyl sulfate (SDS) loading buffer, and then separated by 8% SDS–PAGE and transferred to PVDF membranes. 5% nonfat dry milk in TBST was used for blocking nonspecific blots for 1 h at room temperature. Membranes were then incubated with various primary specific antibodies overnight at 4°C. The membranes were then washed with TBST for three times. After incubation with corresponding horseradish peroxidase-conjugated secondary antibodies for 1 h at room temperature, membranes were washed three times for another 30 min in TBST. Subsequently, the protein bands were detected using an Electrogenerated chemiluminescence (ECL; Pierce Biotechnologies Inc., Rockford, USA) detection reagent (Pierce Biotechnologies Inc., Rockford, USA). The primary antibodies are as follows: TAZ (CST, Danvers, MA, USA), p-Lats1/2 (CST, Danvers, MA, USA), Lats1/2 (CST, Danvers, MA, USA), N-cadherin (CST, Danvers, MA, USA), E-cadherin (Santa Cruz, Danvers, MA, USA), Nanog (CST, Danvers, MA, USA), c-Myc (CST, Danvers, MA, USA), actin and tubulin (Santa Cruz, California, USA), Gelsolin (CST, Danvers, MA, USA).

### Co-immunoprecipitation (Co-IP)

HEK293T cells, transfected with Flag-LMP1 and Flag-LMP1ΔCT plasmids, were lysised with IP buffer containing protease inhibitor cocktails (Roche, Basel, Switzerland). Aliquots of 2000 μg of proteins from each sample were pre-cleared by incubation with 20 μl of protein A/G plus-Agarose (Santa Cruz, Danvers, MA, USA) for 1 h at 4°C. Pre-cleared samples were incubated with the anti-Flag antibody (2 μg/sample) overnight at 4°C. Then 20μl protein A/G plus-Agarose was added into each sample and incubated for 3 h at 4°C. The protein A/G was then washed 4 times with cold IP lysis buffer, boiled with 6× SDS loading buffer and the bound proteins were separated by 10% SDS–PAGE, and transferred onto a PVDF membrane followed by Western blot analysis.

### Immunofluorescence microscopy

Cells were seeded on poly-L-lysine-coated coverslips and fixed with 4% formaldehyde for 15 min. After fixation, cells were incubated with a FITC-conjugated phalloidin (50 mg/ml; Sigma, St. Louis, MO, USA) for 1 h. All coverslips were mounted with the Vectashield reagent (Vector Laboratories Inc., CA, USA) and visualized by confocal microscopy using a Leica DMI 3000B laser-scanning confocal microscope (LEICA, Germany).

### RNA interference

siRNAs against gelsolin and scrambled control were synthesized by Genepharma (Suzhou, China). RNAi Sequences used as follows: gelsolin siRNA, 5′-GGUUGGAAAGGAUUCUCAA-3′ for #1, and 5′-ACAUCAUUCUGUACAACUA-3′ for #2. Cells were transfected with siRNAs using DharmaFECT-1 (Dharmacon, London, British) as described by the manufacturer's instruction. After 12 h, transfection medium was replaced with regular medium and cells were further incubated for 36 hours before further analysis.

### Lenti-virus production and infection

HEK 293T cells were co-transfected with the packaging plasmids pMD2.G and psPAX2, and the shRNA- or ORF-containing vectors. Virus-containing supernatant was collected 48 h and 72 h after co-transfection. Then, virus-containing supernatant were added to the target cells cultured in fresh complete medium containing 5-8 μg/ml polybrene. The target cells were centrifuged at 1,000× g for 30-60 min at room temperature to increase transduction efficiency. Twenty-four h later, the infected cells were selected with 2 μg/ml puromycin (Gibco, New York, USA).

### Fractionation

Fraction of nuclear proteins was isolated using the NE-PER Nuclear and Cytoplasmic Extraction Kit (Thermo, New York, USA) according to the manufacturer's protocol. After fractionation, 50 μg of protein was used for western blot analysis of TAZ in nucleus. Lamin B was used as markers of the nucleus.

### Luciferase assay

YAP/TAZ responsive luciferase reporter (4× GTIIC-lux) plasmid was co-transfected with pRL-SV40 for normalization into control CNE1 and CNE1-LMP1 cells. Renilla luciferase and firefly luciferase activities were measured by using a reporter assay system according to the manufacturer's directions (Promega, Fitchburg, WI, USA). YAP/TAZ responsive luciferase reporter sequence has been described previously [25].

### Immunohistochemistry

The use of the patient samples was approved by Human Ethical Committee of Xiangya Hospital, Central South University. For detection of LMP1 and TAZ, consecutive slide-mounted NPC and gastric cancer sections were first treated with proteinase K at room temperature for 15 min. Endogenous peroxidase activity was inhibited by incubating with 3% H_2_O_2_ (DAKO, Carpinteria, CA, USA). Nonspecific binding was blocked with Antibody Diluent and Background Reducing Component (DAKO, Carpinteria, CA, USA). Sections were then incubated with anti-TAZ (CST, Danvers, MA, USA; 1:50 dilution) and anti-LMP1 (DAKO, Carpinteria, CA, USA; 1:100 dilution) antibodies at room temperature for 1 h. After a washing step, a HRP-conjugated secondary antibody was added and sections were incubated at room temperature for 20 min. Tissue sections were then treated with DAB reagent (DAKO, Carpinteria, CA, USA); 3, 3′-Diaminobenzidine tetrahydrochloride was used as a chromogen. All images were acquired on an Olympus BX51 microscope (Leica, Germany).

### Cell proliferation assay and mammosphere assays

5×10^4^ adherent cells were seeded in six-well plate, and cell numbers were determined by trypan blue exclusion through cell count. Mammosphere assays were performed as previously described [51]. Briefly, single-cell suspensions of cell lines were suspended at a density of 2000 cells/ml in Dulbecco's modified Eagle's medium/F-12 containing 2% B27, 10 ng/mL fibroblast growth factor, and 10 ng/mL epidermal growth factor and seeded into six-well plates with low-attachment surface (1 ml per well). Mammospheres were counted after 1 to 2 weeks.

### Statistical analyses

All the data were processed using SPSS 18.0 (SPSS Inc., USA) and GraphPad Prism (Version 5, GraphPad software, Inc., USA) and presented as mean ± SD, and a P value less than 0.05 was considered to be statistically significant (*P < 0.05, **P < 0.01 and ***P < 0.001).

## SUPPLEMENTARY MATERIALS FIGURES AND TABLES


