# Supplementary material for: Positive regulation of TAZ expression by EBV-LMP1 contributes to cell proliferation and epithelial-mesenchymal transition in nasopharyngeal carcinoma
Source: Oncotarget. 2016 Dec 2;8(32):52333–44. doi: 10.18632/oncotarget.13775 (PMC5581032; doi:10.18632/oncotarget.13775)
Supplement: Supplementary file 1 [file oncotarget-08-52333-s001.pdf]

Positive regulation of TAZ expression by EBV-LMP1 contributes to cell proliferation and epithelial-mesenchymal transition in nasopharyngeal carcinoma

SUPPLEMENTARY FIGURES AND TABLES

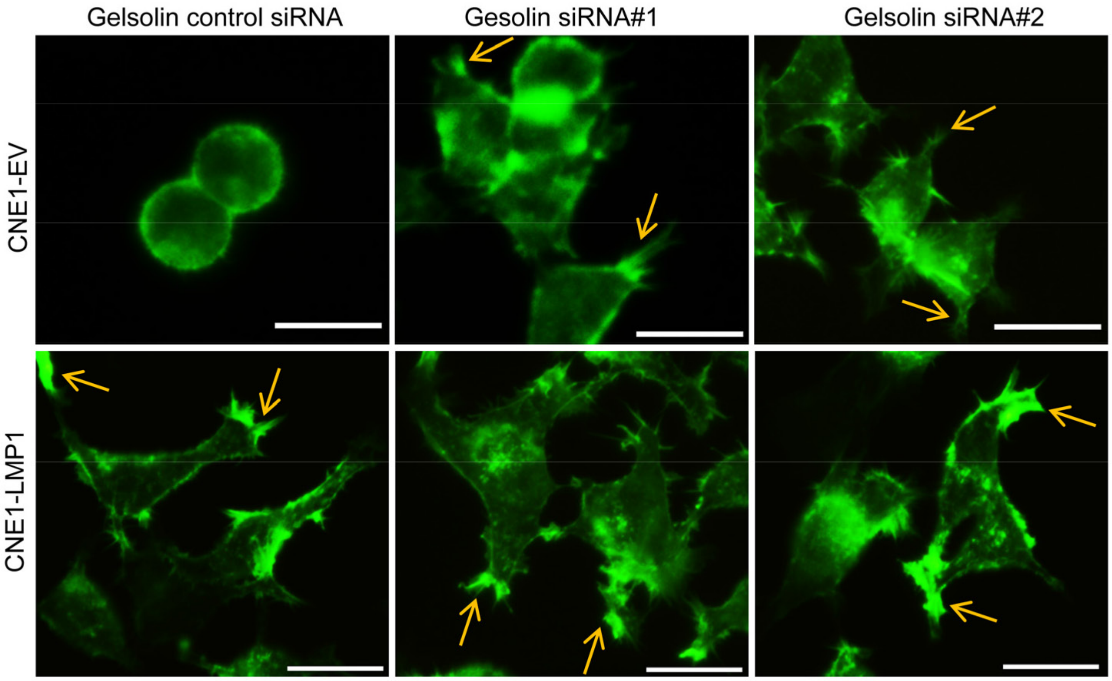

Supplementary Figure S1: Induction of F-actin rearrangement by gelsolin. CNE1-EV and CNE1-LMP1 cells were fixed, and stained with a FITC-conjugated phalloidin (Green) after knockdown of gelsolin. Knockdown of gelsolin led to formation of the microspike-like actin structures (filopodia) at the plasma membrane (arrows). In contrast, Knockdown of gelsolin did not lead to obvious change of actin substructures (microspike-like actin structures) in CNE1-LMP1 cells.

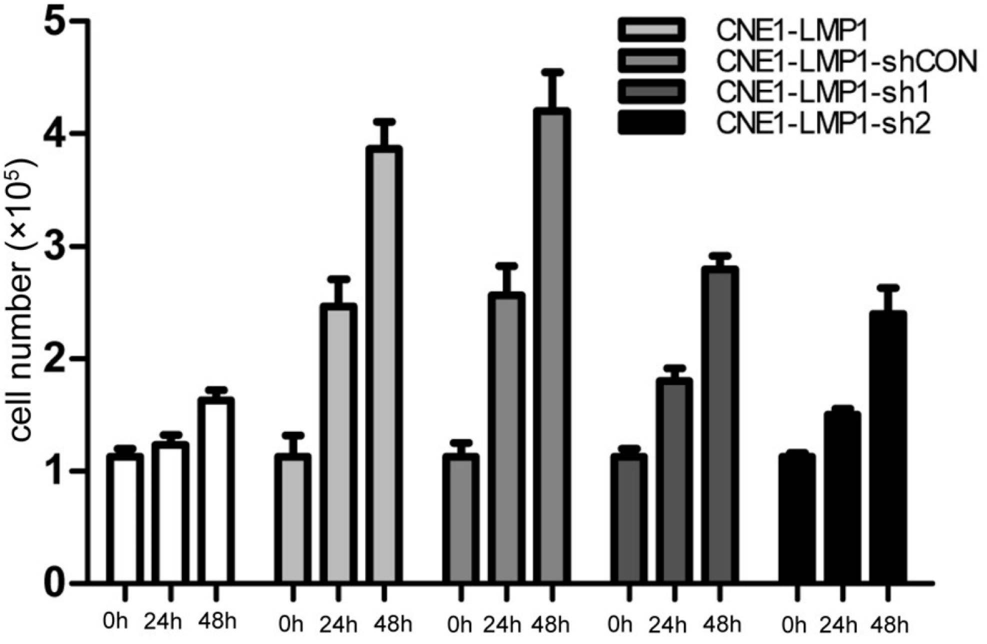

Supplementary Figure S2: Cell counting of CNE1-EV, CNE1-LMP1, CNE1-LMP1-shcon, CNE1-LMP1-sh1 and CNE1-LMP1-sh2 cells at the indicated time points. EV, empty vector; sh, short hairpin RNA; shcon, short hairpin RNA control. All the data are from at least three independent experiments and expressed as mean ± SD.

Supplementary Table S1: Primer sequences

Gene	Forward	Reverse
GAPDH	5′-CGGAGTCAACGGATTTGGTCGTAT-3′	5′-AGCCTTCTCCATGGTGGTGAAGAC-3′
TAZ	5′-CCGGCCGGAGAGTACATGA-3′	5′-CTGGTGATTGGACACGGTGA-3′
YAP	5′-CAACTCCAACCAGCAGCAAC-3′	5′-TTGGTAACTGGCTACGCAGG-3′
CTGF	5′-CAGGCTAGAGAAGCAGAGCC-3′	5′-GTAATGGCAGGCACAGGTCT-3′
Cyr61	5′-CAGGACTGTGAAGATGCGGT-3′	5′-GCCTGTAGAAGGGAAACGCT-3′

Supplementary Table S2: Targeting sequences for shRNA

Gene	Targeting sequences
LMP1	#1: 5′- GCTGTACATCGTTATGAGTGA-3′; #2: 5′-GGTGGACTCTATTGGTTGATC-3′
TAZ	#1: 5′-ACGTTGACTTAGGAACTTT-3′; #2: 5′-AGAGGTACTTCCTCAATCA-3′
